# Integrative and Conjugative Element ICETh1 Functions as a Pangenomic DNA Capture Module in *Thermus thermophilus*

**DOI:** 10.3390/microorganisms8122051

**Published:** 2020-12-21

**Authors:** Alba Blesa, Ignacio Baquedano, Sandra González-de la Fuente, Mario Mencía, José Berenguer

**Affiliations:** 1 Department of Biotechnology, Faculty of Experimental Sciences, Universidad Francisco de Vitoria, 28223 Madrid, Spain; 2 Centro de Biología Molecular Severo Ochoa (CBMSO), Universidad Autónoma de Madrid-Consejo Superior de Investigaciones Científicas, 28049 Madrid, Spain; ibaquedano@cbm.csic.es (I.B.); sandra.g@cbm.csic.es (S.G.-d.l.F.); mmencia@cbm.csic.es (M.M.)

**Keywords:** ICE, conjugation, mosaicity, pangenome, *Thermus*

## Abstract

Transjugation is an unconventional conjugation mechanism in *Thermus thermophilus (Tth)* that involves the active participation of both mating partners, encompassing a DNA secretion system (DSS) in the donor and an active natural competence apparatus (NCA) in the recipient cells. DSS is encoded within an integrative and conjugative element (ICETh1) in the strain *Tth* HB27, whereas the NCA is constitutively expressed in both mates. Previous experiments suggested the presence of multiple origins of transfer along the genome, which could generate genomic mosaicity among the progeny. Here, we designed transjugation experiments between two closely related strains of *Tth* with highly syntenic genomes, containing enough single nucleotide polymorphisms to allow precise parenthood analysis. Individual clones from the progeny were sequenced, revealing their origin as derivatives of our ICETh1-containing intended “donor” strain (HB27), which had acquired separate fragments from the genome of the ICETh1-free HB8 cells, which are our intended recipient. Due to the bidirectional nature of transjugation, only assays employing competence-defective HB27 derivatives as donors allowed the recovery of HB8-derived progeny. These results show a preference for a retrotransfer mechanism in transjugation in ICETh1-bearing strains, supporting an inter-strain gene-capture function for ICETh1. This function could benefit the donor-capable host by facilitating the acquisition of adaptive traits from external sources, ultimately increasing the open pangenome of *Thermus,* maximizing the potential repertoire of physiological and phenotypical traits related to adaptation and speciation.

## 1. Introduction

The concept of conventional horizontal gene transfer (HGT) is being confronted by a panoply of lateral gene exchange mechanisms that defy the classical description of transformation, conjugation, and transduction [1]. Mechanisms based in the transfer of genetic information, protected either by capside-like protomers encoded by degenerated prophages [2] or derived from outer and/or cytoplasmic membranes, allow the long-term maintenance and separate transfer of non-genetically linked genes [3,4]. In contrast, classical conjugation (reviewed in [5]) requires the close contact between two cells, allowing the transfer of large DNA fragments from a donor strain to a recipient one in a one-direction process. Donor strains in this standard process are the hosts for mobile conjugative plasmids (CPs) or integrative and conjugative elements (ICEs), which encode the set of genes involved in DNA processing and replication (Dtr) and in mating pair formation (Mpf). For Dtr, the origin of transfer sequence (*oriT*) in the mobile element is recognized by a relaxase, which cleaves one of the DNA strands and remains covalently bound to its 5′ end. Meanwhile, Mpf involves a type IV secretion system (T4SS) that allows surface recognition and connection to the recipient cell and the subsequent ATP-driven transfer of the translocase-ssDNA nucleoprotein complex to the cytoplasm of the recipient strain. ICEs and CPs have evolved for auto-transfer and propagation within a population, and consequently, they also co-transfer passenger genes integrated within the boundaries of these elements. In addition, CPs sometimes integrate into the chromosome of their host through homologous recombination after the acquisition of a resident insertion sequence. This leads to the generation of “high frequency of recombination strains” or Hfr, where the *oriT* of the CPs is used to start the transfer of chromosomal genes in an ordered fashion, leading to a gradient of transfer frequencies of genes, with a decreasing rate of transfer along distance from the CP integration point [6].

In addition to this classical model of conjugation, alternative mechanisms of conjugation-like processes exist among phylogenetically distant bacterial groups in which the transfer of chromosomal genes can take place from different points in the genome in an apparently simultaneous action, similar to the distributive conjugation described for *Mycobacterium* spp or similar processes described for *Mycoplasma agalactiae*, *Streptomyces* spp, and *T. thermophilus* [7,8,9].

The strain *T. thermophilus* HB27 is a laboratory model for thermophilic bacteria due to its fast growth and highly efficient and constitutive natural competence apparatus (NCA) [10]. Its genome is polyploid with 4–6 copies [11] of a 1.8 Mbp chromosome and a similar copy number of a 0.23 Mbp megaplasmid named pTT27 [12]. In addition to the acquisition of environmental naked DNA (eDNA), a functional NCA in the recipient cells is also required for an unconventional conjugation process named transjugation. Transjugation allows DNA exchange between *T. thermophilus* cells encoding a DNA secretion system (DSS) that drives the push out of the transferred DNA (tDNA), which is concomitantly imported through the NCA of the recipient cell [13]. Interestingly, the DNA transferred by this mechanism escapes to the Argonaute protein surveillance system that degrades 90% of the eDNA acquired by transformation, supporting a major role in HGT in this bacterium [14].

**Figure 1:**
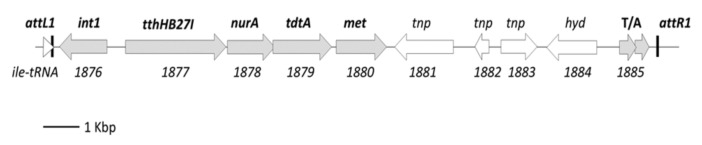
Integrative and conjugative element (ICETh1) structure. A map depicting the genes encoded by ICETh1 is shown. Integration repeats *attL1* and *attR1*, and genes for which biochemical and/or genetic evidences exist regarding their function are labeled in bold and the arrows in gray. The code number of the genes in the chromosome of *T. thermophilus* HB27 is located below the corresponding gene.

In this thermophilic bacterium, the DNA donation trait is linked to the presence of an integrative and conjugative element (ICETh1), which is inserted at the 3′ end of an isoleucine tRNA (chromosome positions 1,778,502 to 1,793,359), which encodes essential components of the DSS [13,15] (Figure 1). However, in contrast to “conventional” conjugative processes in which donor cells cannot receive elements already present in their genome according to the replicon incompatibility principle [5], the presence of ICETh1 does not limit the cell to act as a DNA recipient in mating experiments with other ICETh1 bearing strains [14]. Moreover, transjugation allows the transfer of any gene in the chromosome independently of their localization, showing only minor differences among transfer frequencies [14]. Nonetheless, a 10-fold preference for the ICETh1 itself and for genes encoded by the pTT27 megaplasmid with respect to chromosomal ones has been reported [13,15]. 

Regarding DNA donation in *T. thermophilus*, the most relevant protein of the DSS is TdtA, which is a membrane protein of the FtsK family of DNA translocases that localizes near the cell poles [13]. Its coding gene is co-transcribed with three additional genes encoding a bifunctional restriction-methylation enzyme (TthHB27I) [16], a homologue of nucleases of the NurA family, and a putative methylase (Figure 1). In addition to TdtA, the restriction enzyme TthHB27I and the NurA-like proteins have been shown to be strictly required for DNA donation [13], whereas the absence of the putative methylase develops only minor effects on transfer efficiency [17]. 

The available experimental data commented above lead to hypothesize a model for transjugation in which the restriction enzyme TthHB27I could cleave the donor DNA at different locations in the polyploid genome, whereas TdtA, putatively helped by NurA, could use these cuts as origins for transfer to the recipient strain. A concomitant need for DNA repair of the cleaved sites in the donor cell could underlie the requirement for HepA (TTC0147), which is a homologue of TdtA belonging to the HerA family of archaeal DNA helicases involved in DNA repair [18]. According to this model, recipient cells would receive different genome fragments from the donor cells simultaneously during transjugation, leading to the generation of genomic mosaicism among the progeny after integration by recombination, which is similar to those described for *Mycobacterium* spp [8] or *Mycoplasma agalactiae* [19].

In this work, we check this hypothesis by applying whole genome sequencing (WGS) analysis to progeny clones derived from mating experiments between the ICETh1-containing strain HB27 and the ICETh1-free HB8 strain, which syntenic genomes show enough single nucleotide polymorphisms’ (SNP) density as to allow an accurate parenthood analysis [20]. The results obtained support the hypothesis of multiple origins of transfer working simultaneously but also revealed that most of the progeny derives from the ICETh1-containing HB27 strain that supposedly captures genes from the ICETh1-free HB8, which is intended as a recipient strain in our hypothesis model. This implies the existence of genetic retro-transfer upon mating and supports a role of ICETh1 as a gene-capture element, likely providing selective advantages to the host cell.

## 2. Materials and Methods 

### 2.1. Strains and Growth Conditions 

The strains of *T. thermophilus* used in this work are described in Table 1. *T. thermophilus* cells were grown at 60 °C in TB (*Thermus* broth, Trypticase 8 g/L, Yeast extract 4 g/L, NaCl 3 g/L in carbonate-rich mineral water) under rotational shaking (150 rpm). For plates, 2% agar (*w*/*v*) was used. Kanamycin (Kan, 30 µg/mL) and/or hygromycin B (Hyg, 100 µg/mL) were added for selection when needed. 

### 2.2. Transjugation Assays

*T. thermophilus* HB27 and HB8 parental strains employed in mating experiments [23] carried a gene cassette encoding a thermostable resistance to Kan (*kat*) or Hyg (*hph*) inserted into the chromosome (CK and CH) or in the megaplasmid (PH) (Table 1). Briefly, mates were grown for 16 h in TB with the respective selection antibiotic. Then, 0.1 mL of each culture were mixed in the presence of 5 units of DNAse I (Roche, Basel, Switzerland), washed by centrifugation in 1 mL of TB, re-suspended in 10 μL of TB also with DNAse I (5 units, Roche), and spotted onto a nitrocellulose filter (0.22 μm, GSWP, Millipore, Burlington, MA, US) laid on pre-warmed TB agar plates. Plates were incubated for 4.5 h at 60 °C, and the cells on the filters were detached by soaking and shaking in 1 mL of TB, and further serially diluted for plating on TB agar plates amended with Kan and Hyg. 

### 2.3. Whole Genome Sequencing (WGS)

Individual transjugant clones from the mating experiments, resistant to both selection antibiotics (Kan and Hyg), were re-streaked and grown in a second selection plate to guarantee clonality. Overnight cultures of 20 mL were submitted to DNA extraction. Analogously, parental strains were grown in its respective selection antibiotic and genomic DNA was extracted in parallel, employing the DNeasy Blood & Tissue kit (Qiagen, MD, Germantown, USA), following manufacturer’s instructions for Gram-negative bacteria. RNase A (Roche) was added to all the samples. After quality control check, samples were subjected to WGS at MicrobesNG (Birmingham) using Illumina next-generation sequencing (Miseq, read type 2 × 36-251). The reads were trimmed using Trimmomatic [24], and quality sequence analyses were performed over reads using FastQC software [25]. Coverages of the sequences were calculated with the GenomeCoverageBed tool (http://bedtools.readthedocs.io/en/latest/content/tools/genomecov.html). They ranged from 31× for transjugant 11 to 174× for transjugant 5. Assemblies were provided by the sequencing center rendering from 697 to 910 contigs for transjugants 2 and 3, respectively. Similar coverage and contigs number were obtained for the parental strains. 

### 2.4. Parenthood Analysis 

Reference genomes for *T. thermophilus* HB27 (NC_005835.1, NC_005838.1) and HB8 (NC_006461.1, NC_006462.1, NC_006463.1, AB677526.1) strains were downloaded from the NCBI. The reads of parental and transjugant strains were aligned against the reference genome using BWA aligner [26]. Picard Tools (https://broadinstitute.github.io/picard/) was used to clean, sort, and mark duplicates of mapped BAM (Binary Alignment Map) files. The final BAM file was used for Variant Calling [27]. This was performed using the GATK toolkit [28] to identify SNPs on each transjugant with the HaplotypeCaller tool. The annotation of the variants was carried out with snpEff software [29]. The annotation output files in native VCF (Variant Calling Format) were processed using an in-house script written in Perl language (parser_gatkSnpeff_2xls.pl) to obtain an excel-like annotation file. The results were processed using an in-house script written in Python language (compareSNP_betweenSamples.py) to compare all variants positions between samples and to detect the genetic location of each variant. Variants found in the parental strains with respect to the reference genomes in the Genbank database were disregarded, as they were either the consequence of strain domestication or the products of sequence errors in these Genbank databases. Circos software [30] was finally used for the visualization of the SNPs data in a circular layout.

### 2.5. Identification of Non-Homologous Genes Acquisition 

The location of the resistance gene cassettes in the transjugant strains was assessed through the alignment of the corresponding reads against the *kat* [31] and *hph* [32] sequences by using the LAST software (http://last.cbrc.jp/). Overlapping reads to the 5′ and 3′ extremes of the respective gene cassettes were used to search by BLAST in the reference genomes, thus leading to their precise localization. The search for HB8- and HB27-specific genes in the transjugants was carried out by using as reference fasta files generated by comparison of the reference genomes. These files of parental-specific genes were further used to search by BLAST for matches among the contigs of the transjugants, using for this a 40% of minimal sequence cover and an *e*-value = 0 to match identical sequences.

## 3. Results

### 3.1. Selection of T. thermophilus Strains for Parenthood Analysis

The genome of *T. thermophilus* HB27 consists of 4–6 copies of a 1,894,877 bp chromosome and a similar copy number of a 232,605 bp megaplasmid named pTT27 [11]. This strain can act as a donor in transjugation due to the presence of ICETh1 [13,15]. For parenthood analysis, we selected the phylogenetically related *T. thermophilus* HB8 strain as a receptor partner. *T. thermophilus* HB8 is also polyploid [33], with a similar copy number for its chromosome (1,849,742 bp) and several episomal elements. It has a 256,992 bp megaplasmid with 72% of the genes conserved in HB27 pTT27 [20] and two other plasmids absent in HB27: a large plasmid related to biofilm formation (pVV8, 81,151 bp) present only in old stocks of this strain [34], including the ones used in our assays, and a small 9322 bp plasmid (pTT8) coding for yet unknown traits. The high degree of conserved genes (1860 genes, 93% of total), the synteny between the two strains [20], and the presence of a relatively high frequency of differential SNPs (1/124 bp along the whole genome) supported the consideration of the HB27/HB8 mating pairs as an excellent source of data for high-resolution parenthood analysis. A lesser genomic conservation (72% of genes, 181 genes) was detected among the respective pTT27 megaplasmids but still with enough synteny conservation to allow the analysis. Alignment of the genomes of the two strains revealed the existence of 8–9 SNPs per kbp between the conserved genes, regularly distributed along the genome of both strains, so as to identify their origin in parenthood analysis. As mentioned above, the genes encoded within the ICETh1 are among the set of HB27-specific genes, as this DSS coding mobile element is not present in the HB8 strain. Previous experimental data confirmed the fact that by itself, the DNA donor capacity of HB8 is negligible [13]. Therefore, we set HB8 to act as the recipient strain of genes transferred from an HB27 donor in the mating experiments.

### 3.2. Transjugation between T. thermophilus HB7 and HB8 Strains

A chromosome-labeled Kanamycin resistant (Kan^R^) HB27 derivative (CK1) was used as “donor” and two Hygromycin resistant (Hyg^R^) HB8 derivatives were used as “recipient” strains, which were labeled either in the chromosome (CH81) or in the pTT27 megaplasmid (PH81) (Table 1). None of the marked strains showed any detectable phenotypic difference with the corresponding wild-type strain at the level of growth or morphology in TB medium. From around five hundred clones resistant to both antibiotics (transjugants), eight were sequenced: five derived from CK1 mating to the PH81 strain (T1–T5), and three from mating CK1 with the CH81 strain (T6-T8).

Sequence recruitment of the parental strains CK1, CH81, and PH81 with their respective reference HB27 and HB8 genomes in Genbank (Table 2) confirmed the expected high percentages of identity (>98%), whereas sequence recruitment of the CK1 parental (derived from HB27) to the HB8 reference genome rendered lower percentages (CK1 > HB8 reference = 87.53%) and, reciprocally, sequence recruitment of the CH81 and PH81 parentals (derived from HB8) to the reference HB27 genome also yielded lower similarities (CH81 > HB27 reference = 79.46%), as expected (Table 3). However, in contrast to our expectations, the sequences of the 8 transjugant strains were much better recruited to the HB27 reference (>97.8%) than to HB8 reference (<87.9%), as these percentages were very similar to those obtained for the alignment of HB27-derived CK1 strain genome to that of HB27 (98.45%) and HB8 (87.53%) reference genomes, respectively. These results clearly show that the CK1 strain has acted as recipient of the *hph* resistance gene (allowing its selection with hygromycin) coming from the HB8-derived PH81 and CH81 strains. Thus, we concluded that the ICETh1-containing HB27 strain, initially intended as donor, acted as a gene(s) recipient from the ICETh1-free strain, which was initially set as the recipient.

### 3.3. Selection of HB8-Derived Transjugants

In order to allow only an unidirectional transfer of genes from HB27 to HB8 derivatives, we carried out a similar transjugation experiment but with the CK2 strain, a HB27 derivative in which natural competence has been abolished due to a full deletion of the pilin-encoding *pilA4* [14], which is thus unable to act as a recipient cell in transjugation (Table 1). As before, this CK2 strain was mated with CH81, the chromosome-labeled HB8 derivative. Five individual transjugants, thereafter named T9 to T13, were selected from the transjugants population obtained in this mating experiment (around two hundred), and subjected to WGS as above, with mean sequence coverages ranging between 31 (T11) and 145 (T12)-fold. Further sequence recruitment analysis revealed the expected higher match of their sequences to the HB8 reference genome (>99%), whereas recruitment to the HB27 reference genome ranged between 81.46% (T13) and 84.37% (T10) (Table 3). These data confirmed the nature of the T9 to T13 clones as derivatives of the HB8 strain. 

### 3.4. Single Nucleotide Polymorphisms (SNPs) Reveal Parenthood of Genes

The “variants calling” analysis revealed the existence of SNPs between our sequenced parental strains and the respective reference genomes: 55 and 111 in the chromosome of the HB27 and HB8 parentals and 7 and 3 for their respective pTT27 megaplasmids. These SNPs could correspond to laboratory adaptation modifications among the stocks of the wild-type strains used to isolate the corresponding antibiotic-labeled derivatives, or to sequence errors in the reference genomes that were obtained by older methods with lesser coverage [12]. Whatever the case, as our interest was to compare the sequences of the transjugants, we used the sequence of our parental strains CK1, CK2, CH81, and PH81 as reference. Cross-comparison between these parental reference sequences revealed the existence of 14,818 and 2167 SNPs specific to the C/PH81 parental (HB8-derived) compared to the CK1 strain (HB27-derived) located in the chromosome and in the pTT27 megaplasmid, respectively (Table 3). 

The presence in the T1 to T8 transjugants of SNPs specific to the reference C/PH81 parental was subsequently used as indicative of the transfer and integration of HB8-derived genes in the HB27-derived CK1 strain. By following this comparison, a range from 5 to 114 HB8-specific SNPs was detected in the chromosome of the transjugants, whereas from 0 to 283 HB8-related SNPs were identified within the pTT27 megaplasmids (Table 3).

**Figure 2:**
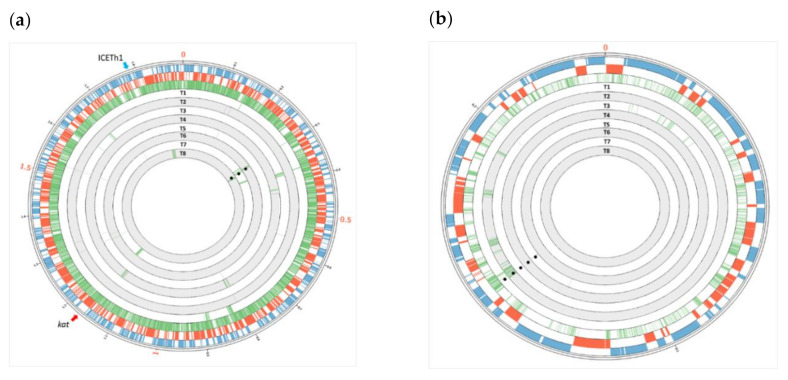
(**a**) Chimeric nature of the chromosome of retrotransjugants derived from HB27. External blue and red circles indicate the positions of forward and reverse annotated ORFs in the chromosome of the HB27 strain, respectively. Green bars in the first internal circle indicate the positions of the single nucleotide polymorphisms (SNPs) present in the chromosome of the HB8 strain. Underlying concentric circles correspond to the genome of transjugants T1 to T8 from the outermost to the innermost circles, respectively. Presence of green lines denotes SNPs acquired from the HB8 strain back into an HB27 background genome. (**b**) Chimeric nature of the pTT27 megaplasmid of retro-transjugants derived from HB27. External blue and red circles indicate the positions of forward and reverse annotated ORFs in the pTT27 megaplasmid of the HB27 strain, respectively. Green bars in the first internal circle indicate the positions of the SNPs present in the pTT27 megaplasmid of the HB8 strain. Underlying concentric circles correspond to the genome of transjugants T1 to T8. The presence of green lines denotes SNPs acquired from the HB8 strain back into an HB27 background megaplasmid. Position of the *kat* gene is labeled with a red arrow and the *hph* gene is labeled in black dots. The position of ICETh1 is labeled as a blue arrow.

The distribution of SNPs along the transjugants (T1–T8), depicted as green lines in the internal circles of Figure 2, revealed the acquisition and integration of genes along different genome regions in the individual transjugants (Figure 2 a). In all cases, the *hph* marker from the C/PH81 parental was integrated by homologous recombination at the expected site in all the transjugant clones: In T1 to T5, the marker was integrated at loci TT_P0146 (positions 148,362 to 14,9594) in the megaplasmid, homologue to the HB8 loci TTHB198 (89% of identity), where the marker was inserted in the PH81 strain. Concomitantly, in clones T6, T7, and T8, the *hph* marker was localized at the chromosomal loci TT_C0313 (positions 299,800 to 301,557), homologue to chromosomal loci TTHA0672 (99% of identity), where the marker was inserted in the CH81 strain.

In addition to the presence of the *hph* marker at the expected megaplasmid site in transjugants T1 to T5, transjugants T2 and T3 contained additional insertions of HB8 DNA in other regions of the megaplasmid (Figure 2 b), which are approximately defined by the position of boundary SNPs (Table 3 and Table S1). Clone T2 showed that the transferred DNA was distributed along five patches of 645, 894, 2401, 2699, and 5164 bp in the megaplasmid, which are identified by a total of 283 SNPs. Transjugant T3 also showed 51 SNPs in the megaplasmid distributed along six different DNA regions, which were transferred from the HB8-derived parental strain (1 single SNP; 85, 342, 1558, 2328, 2960 bp). In contrast, no SNPs were identified within the pTT27 megaplasmid of T4, for which only the presence of the *hph* marker was detected, and signals of the transfer at two different genomic sites were detected for T5 embracing five SNPs, one of them corresponding to the region where the selection marker was located. 

Interestingly, transjugants T1 to T5 also showed HB8-specific SNPs in different regions of the chromosome (Figure 2a and Table S1). Clone T1 showed 109 SNPs clustered along six separate regions of the chromosome, four of them of small size (from a single SNPs to 1432 bp) and the other two, larger sizes (10,771 and 12,403 bp) (Table 3). Transjugant T3 presented several (65) SNPs corresponding to the acquisition of HB8 genes at six different chromosomal regions, three small ones (2 single SNPs and a 60 bp fragment) and three longer ones (2226, 6346, and 7201 bp). In addition, clone T4 showed a broad transfer of chromosomal genes of different sizes (718, 791, 899, 3032, 3056, 11,071 bp) into six different regions, involving 92 SNPs. In contrast to this extensive co-transfer of chromosomal genes, the transjugant strains T2 and T5 showed a reduced number of SNPs (5–6) but scattered along the chromosome. 

Analysis of transjugants T6, T7, and T8, grown from CK1 X PH81 mating, revealed the presence of the *hph* marker at the homologous chromosome loci and SNPs in genes around it in three of them. In addition, the presence of genes along other regions of the chromosome was detected. Clone T6 included SNPs corresponding to recombination at two separate chromosomal loci far away from the inserted marker. Transjugant T7 contained 114 SNPs distributed along 11 loci across the chromosome, two of them of 648 and 1915 bp, corresponding to flanking regions around the selection marker, whereas others were located at longer distances in the chromosome, ranging from single SNPs to large sequences of up to 12 kbp. Finally, clone T8 showed a single 19.5 kbp segment insertion far from the selection marker. However, no signal of recombination events with genes from the megaplasmid could be detected in T6, T7, or T8 clones.

**Figure 3:**
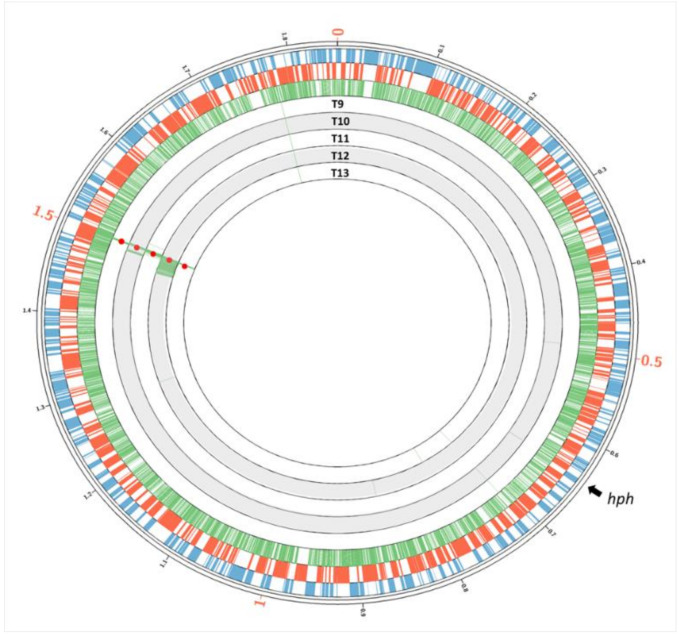
Chimeric nature of the chromosome of transjugants derived from HB8. External blue and red circles indicate the forward and reverse annotated ORFs in the chromosome of the HB8 strain, respectively. Green bars in the first internal circle indicate the positions of the SNPs present in the chromosome of the HB27 strain. Underlying concentric circles correspond to the genome of transjugants T9 to T13. The presence of green lines denotes SNPs acquired from the HB27 strain. Positions of the *kat* and *hph* genes are labeled in red dots and black arrow, respectively.

The variants’ calling analysis of transjugants T9 to T13, derived from the unidirectional transjugation from CK2 (HB27 derivative, impaired in NCA and thus only able to act as donor) to CH81 (HB8 derivative), was carried out in a similar way—in this case, specifically looking for HB27-specific SNPs that would had been transferred to HB8 cells (Table 4). In these T9–T13 transjugant strains, most transferred genes were found adjacent to the *kat* marker (TTHA_1576 in HB8, positions 1,498,261–1,499,534), with recombinant boundaries ranging from 1.9 (T9, T13) to 30.8 (T12) kbp. Beyond these transferred regions, only small fragments and punctual sequences of individual HB27-specific SNPs were detected (Figure 3), and no detection of massive genetic exchange among megaplasmid genes was detected in any of the cases.

### 3.5. Transfer of Parental-Specific Genes

The parenthood analysis described above for the transjugants T1 to T8 was performed on clones obtained from mating experiments between competence proficient strains; thus, both are able to acquire DNA transferred by transjugation. Our results clearly showed that fragments of the “recipient” strain had been sent back to the intended donor and integrated into its chromosome, which was likely through homologous recombination. In order to know if, in addition to these homologous genes, other non-homologous HB8 specific genes were transferred too, a different approach was carried out. For this, we performed a search for ORFs present in the parental HB8 but not in the HB27 references (Materials and Methods). This search identified 183 HB8-specific genes: 66 HB8-specific genes encoded within the chromosome, 56 HB8-specific genes in the pTT27 plasmid, all the 59 genes present in megaplasmid pVV8, as well as the 8 genes from the small pTT8 plasmid, the last two elements absent in HB27 (Table S2). Then, we used the corresponding coding sequences of these HB8-specific genes as targets for recruitment analysis on the raw sequences of each transjugant. No transference of HB8-specific genes was detected for transjugants T4, T5, T6, and T7. However, in T1 and T2 clones, putative transposases of the ISTh4 family, with several copies across the HB8 genome but absent in HB27 [9,35], were found integrated at the chromosomal gene TTC0904 in the T1 strain and in a non-coding region near the megaplasmid gene TTP0147 in clone T2. In the latter case, integration could have been the consequence of homologous recombination, as the integration point is flanked by SNPs of the HB8 parental strain. However, integration into TTC904 in the T1 strain seems to be the consequence of a genuine transposition event, as flanking genes showed no signal of integration of HB8 counterpart genes.

In addition to this, HB8-specific genes TTHA0285 and TTH0A286 encoding a hypothetical protein and a putative serine protease, respectively, were found inserted between the HB27 genes TTC1700 and TTC1701 in clone T3. As above, this seems to be the consequence of recombination events with homologues of the latter genes, acting as recombination arms in a genetic organization similar to that found in the HB8 strain. In addition, in transjugant T3, a gene annotated as putative transcription factor (TTHB073) but likely more related to transposases, interrupts the megaplasmid gene TTP030. Finally, the HB8 chromosomal gene TTHA498 (coding for a hypothetical protein) appears integrated in the genome of clone T8, although the small size of the corresponding contig did not allow us to accurately localize it. 

In conclusion, in addition to the acquisition of homologues of genes already present in the HB27 strain, the retro-transfer and integration of non-homologous genes from the theoretical recipient strain was detected, including complete insertion sequences of the IS4 family, contributing to the observed genetic mosaicism of the transjugants.

A similar analysis was carried out in transjugants T9 to T13, revealing the presence of genes specific to HB27 in three of the transjugant strains (Table S3), including sequences identical to the ICETh1-encoded translocase TdtA in clone T9, the gene TTC0952 encoding a putative transporter in clone T10, and genes TTC0398 and TTC0857 in clone T12, annotated as genes encoding a transcription repressor and a membrane protein, respectively.

## 4. Discussion

In this article, we have tested the hypothesis that proposes transjugation as a process involving the simultaneous transfer of DNA from different points in the genome of a donor cell to the genome of a recipient counterpart. In addition, as shown in this work, we observe that the transjugation process in *T. thermophilus* can easily lead to bidirectional transfer, increasing the odds of genetic shuffling and genomic rearrangements, ultimately keeping in a continuous update the Thermus pangenome. We employed two strains with high genomic synteny, one—HB27—carrying the ICETh1 that provides DNA donor functions in transjugation, and the other—HB8—devoid of it and therefore expected to act as DNA recipient, being both of the strains active in natural competence. Our data support a role of ICETh1 as a mobile element conferring DNA scavenging capacity to its host. The main data supporting this view are discussed below.

### 4.1. Detection of Retrotransfer

Our current model for transjugation proposes that the activity of the TthHB27I restriction–methylation enzyme encoded by ICETh1 generates the substrates used by the TdtA DNA translocase for secretion and subsequent transfer to the recipient cells in a way that could have consequences similar to the “distributive conjugation” described for *M. smegmatis*. In this mycobacterial process, different regions from the genome are transferred with similar efficiencies in an apparently random way from a donor to a the recipient cell in an unidirectional way through a yet poorly known mechanism, leading to progeny that provide evidence of recipient cells containing fragments of DNA from the donor in different regions of the genome [22]. As it happens with *Mycobacterium*, in *T. thermophilus,* the transfer frequencies of different regions of the chromosome are similar, supporting random transfer from different origins in the genome [14,36]. Using a similar strategy, we took advantage of strain-specific SNPs to distinguish between the origin of the transferred genes in the progeny. We actually found genomic mosaicity among the progeny in a single mating experiment, with eight isolates containing a diversity of parental fragments within their genomes. However, in contrast to our expectations of getting a recipient genome with a set of genes from the donor spread in the genome, as it happens in *Mycobacterium*, we got derivatives of the so-called “donor” strain containing a mosaic of genes coming from the intended “recipient” strain. Therefore, during incubation time along which both parental strains were in contact on a filter in the presence of DNase, the ICETh1 was hypothetically transferred to the HB8 recipient, where it expressed the DNA donation system (DSS), allowing for the transfer back from HB8 to the initial HB27 donor of different regions of the recipient genome. Although the ability of the HB8 strain to function as a donor in transjugation upon acquisition of the ICETh1 was already known [13], the short time needed for the process was not expected. This retro-transfer of genes back to the donor has also been described for conjugative processes that take place in *Mycoplasma agalactiae* in which the presence of a small ICE-like element in the donor strain leads to the retro-transfer of genes from different genomic regions of the ICE-less recipient, and so, generating genome mosaicity in the donor [19,37]. However, in the case of *T. thermophilus*, it is possible to set the direction of the transfer by using mutants of the donor cell lacking NCA and thus, the capability to uptake DNA, leading to the isolation of recipient derivatives only (transjugants T9-13). Therefore, our data support that after mating experiments between wild-type HB27 and HB28 strains, only a minor fraction of the transjugants probably derive from the recipient, whereas most of the progeny derives from the donor, showing different degrees of mosaicity within their genomes (see below). As the number of clones sequenced (eight) was relatively low, it is not possible at this point to establish the relative proportion between both classes of progeny. On the other hand, the reason underlying this bias in favor of donor-derived strains can only be speculated, and a faster growth rate on selective medium of the donor, or its much higher transformation efficiency (>10 fold) compared to HB8 could, at first, generate the observed over-representation of transjugants derived from HB27. The presence of a specific restriction enzyme in HB8 (TthB8I) [38] could also play a role in limiting the entrance of DNA in this HB8 host and decrease the frequency of HB8-derived transjugants.

### 4.2. Mosaicity of the Progeny

Independently of the retro-transfer of genes, the detailed analysis of the presence of strain-specific SNPs in the progeny clearly shows genomic mosaicity, with genes acquired from genomic regions far from those selected by the antibiotic resistance, even including genes located in different replicons (Figure 2). The transferred DNA was integrated, sometimes as large regions of several kbp (up to 30 kbp insertions were detected), whereas, in other cases, the mosaicity is apparently the consequence of micro-recombination processes that involve only one or two SNPs. Some of the large insertions carry non-homologous genes inserted between homologous ones that potentially function as recombination arms, as in the case of genes TTHA0285 and TTH0A286 integrated in clone T3 by recombination with the conserved TTC1700 and TTC1701 genes. Interestingly, in the analyzed derivatives of the HB8 strain (T9-13), the large regions transferred correspond only to the boundaries of the selection marker, whereas only a few additional SNPs from the donor were found along the genome. In contrast, retro-transfer detected in HB27-derived T1 to T8 strains involve large DNA fragments spread all over the genome, pointing to a greater tolerance of the HGT barriers in the HB27 donor respect to the HB8 recipient strain.

### 4.3. A Putative Mechanism for Retro-Transfer

Our current hypothesis regarding the mosaicity detected among the transjugants derived of HB27 vs. HB8 matings suggest a mechanism in which the ICETh1 is transferred to the HB8 strain, where, instead of being integrated into its target site (actually present in the HB8 strain), it expresses the DSS operon that, among others, includes the bioactive restriction–methylation enzyme TthHB27I [16], which is an unconventional enzyme showing a double function and whose restrictase activity depends on SAM (S-adenosyl-methionine). We hypothesize that this enzyme generates cuts at several positions of the recipient strain that can be further used as substrates processed by TdtA [22]. The activity of this translocase would generate the retro-transfer of the genetic information to the original HB27 host and the further integration of the genes in the chromosome, either by homologous recombination or, if an active insertion sequence is involved as observed in clone T2, by transposition. In favor of this hypothesis is the fact that at least part of the *tdtA* gene has been identified in one of the five HB8 clones sequenced (T12), isolated from the mating between HB27 competent-less mutants and the competent HB8 strain, although the present analysis does not allow distinguishing whether TdtA has been integrated or kept as an independent replicon after being transferred to the recipient HB8 strain. Therefore, in the experimental system assayed, the ICETh1 functions as a DNA capture element that could facilitate the adaptation to the conditions of a new environment by taking advantage of the genes encoded and captured from the resident *Thermus spp* strains. Such a retro-transfer capability has been also described in other cases similar to that of ICEST3 from *Streptococcus thermophilus,* which is capable of capturing other MGE from the recipient partner [39]. However, ICETh1 behaves in a way more similar to that of MICE (*Mycoplasma* integrative and conjugative elements), whose transfer from the MICE^+^ donor to the MICE^-^ recipient is proposed to be followed by extensive replication and transposase-mediated random integration into the chromosome, with MICE’s activity as *oriT,* underlying the mobilization of different chromosomal regions [19]. In the case of ICETh1, its site-specific insertion [15] or even its putative maintenance as episome would not be an obstacle for its ability to trans-mobilize chromosomal regions of the recipient cell back to the donor.

## Figures and Tables

**Table 1:** Strains of *T. thermophilus* used in this work.

Strain	Genotype/Resistance	Use	Reference
CK1	HB27 derivative, TTC1211(*gdh1)::kat*/Kan^R^	Parental	[21]
CK2	HB27 derivative, TTC1211(*gdh1*)::*kat*, Δ*pilA4*/Kan^R^	Competence deficient	[22]
CH81	HB8 derivative, TTHA0672::*hph*/Hyg^R^	Parental	This work
PH81	HB8 derivative, TTHB198::*hph*/Hyg^R^	Parental	This work

**Table 2:** Reads recruitment vs. reference genomes. The genomes of the *T. thermophilus* HB8 and HB27 strains were used as reference for the recruitment of sequences obtained for the parental strains and the transjugants in experiments between labeled wild-type HB27 and HB8 strains (T1 to T8) or between a transformation deficient HB27 and a wild-type HB8 strain (T9–T13).

Strain	% Align vs. HB27 Reference	% Align vs. HB8 Reference
CK1	98.45	87.53
C/PH81	79.46	99.70
T1 ^1^	98.54	87.90
T2 ^1^	98.76	87.89
T3 ^1^	98.14	86.49
T4 ^1^	97.87	85.39
T5 ^1^	98.80	87.22
T6 ^2^	98.93	87.09
T7 ^2^	98.73	85.96
T8 ^2^	98.48	85.45
T9 ^3^	82.19	99.54
T10 ^3^	84.76	99.53
T11 ^3^	82.22	99.51
T12 ^3^	83.63	99.52
T13 ^3^	81.46	99.19

^1^ Mating CK1 × PH81; ^2^ Mating CK1 × CH81; ^3^ Mating CK2 (ΔpilA4) × CH81.

**Table 3:** Parenthood analysis of transjugants T1–T8. Number of specific single nucleotide polymorphisms (SNPs) found both in the HB8 strain and transjugants T1 to T8 with respect to the HB27 chromosome and megaplasmid reference and number of regions transferred.

Strain	SNPs vs. HB27 Reference	Nº of Chromosome Regions Involved ^1^	SNPs vs. pTT27 Reference	Nº of pTT27 Regions Involved ^1^	HB8-Specific Genes Detected
CK1	0 ^2^	-	0 ^2^	-	-
P/CH81	14,818	-	2167	-	-
T1	109	6	31	1 ^1^	ISTh4
T2	6	6	283	5 ^1^	ISTh4
T3	65	6	51	6 ^1^	TTHA0285 TTHA0286 TTHB073
T4	92	6	0	1 ^1^	-
T5	5	5	5	2 ^1^	-
T6	39	5 ^1^	0	0	-
T7	114	11 ^1^	0	0	-
T8	50	2 ^1^	0	0	TTHA498

^1^ includes marker location and are defined as different when separated by more than 1 kbp; ^2^ Set to 0 for comparison with transjugants.

**Table 4:** Number of specific SNPs found both in parental strain CK1 and in transjugants T9 to T13 respect to the CH8 chromosome and pTT27 megaplasmid.

Strain	HB8 Reference	Chromosome Regions Involved ^1^	pTT27 (HB8) Reference	pTT27 Regions Involved ^1^	HB27-Specific Genes Detected
CH8	0	-	0	-	-
T9	16	2 ^1^	0	0	*tdtA*
T10	51	3 ^1^	0	0	TTC0952
T11	36	2 ^1^	0	0	
T12	168	2 ^1^	0	0	TTC0398 TTC0857
T13	14	2 ^1^	0	0	-

^1^ includes marker location.

## Data Availability

The original reads of all the strains sequenced in this work have been deposited at the European Nucleotide Archive with the reference PRJEB23540.

## Supplementary Materials

The following are available online at https://www.mdpi.com/2076-2607/8/12/2051/s1, Supplementary Table S1: Transfer of DNA regions between parental strains in the sequenced transjugants, Supplementary Table S2: Strain-specific genes encoded in *T.thermophilus* HB8, Supplementary Table S3: Strain-specific genes encoded in *T.thermophilus* HB27.

## Originality-Significance Statement

Unconventional mechanisms of conjugation are more common than originally though. We demonstrate mosaicity in the progeny after one of these unconventional processes named transjugation that takes place between different strains of *Thermus thermophilus*. We unexpectedly show that the presence of an integrative element coding for the DNA donation capability produces the capture of DNA from the mating partner by retrotransfer, thus providing the host with higher evolvability.
