# Supplementary material for: Integrative and Conjugative Element ICETh1 Functions as a Pangenomic DNA Capture Module in Thermus thermophilus
Source: Microorganisms. 2020 Dec 21;8(12):2051. doi: 10.3390/microorganisms8122051 (PMC7767461; doi:10.3390/microorganisms8122051)
Supplement: Supplementary file 1 [file microorganisms-08-02051-s001.pdf]

Supplementary material

Supplementary Table S1. Transfer of DNA regions between parental strains in the sequenced transjugants. The positions of the regions transferred respect to the indicated reference are defined by the presence of the HB8-specific (T1 to T8) or HB27-specific (T9 to T13) progeny clones. .

Transjugant	Reference	Start	End	Size[1]	Marker
T1	HB27 Chrom	148258	148928	670	
	HB27 Chrom	298094	298599	505	
	HB27 Chrom	370824	372256	1432	
	HB27 Chrom	820717	831488	10771	
	HB27 Chrom	881382	893785	12403	
	HB27 Chrom	1525696	1525696	0	
	HB27 pTT27	150470	152237	1767	*hph* down
T2	HB27 Chr	117837	117837	0	
	HB27 Chr	129078	129078	0	
	HB27 Chr	801112	801112	0	
	HB27 Chr	1285225	1285225	0	
	HB27 Chr	1315683	1315683	0	
	HB27 Chr	1525696	1525696	0	
	HB27 pTT27	149432	154667	5235	*hph* down
	HB27 pTT27	158435	159080	645	
	HB27 pTT27	161097	163796	2699	
	HB27 pTT27	177529	179930	2401	
	HB27 pTT27	192396	193290	894	
T3	HB27 Chrom	367733	374079	6346	
	HB27 Chrom	376442	378668	2226	
	HB27 Chrom	565964	565964	0	
	HB27 Chrom	1525696	1525696	0	
	HB27 Chrom	1614926	1614986	60	
	HB27 Chrom	1661130	1668331	7201	
	HB27 pTT27	8268	11228	2960	
	HB27 pTT27	19175	21503	2328	
	HB27 pTT27	23243	24801	1558	
	HB27 pTT27	31254	31596	342	
	HB27 pTT27	33885	33970	85	
	HB27 pTT27	199126	199126	0	
T4	HB27 Chrom	416296	417195	899	
	HB27 Chrom	419129	419920	791	
	HB27 Chrom	430622	431340	718	
	HB27 Chrom	793578	796610	3032	
	HB27 Chrom	803550	806606	3056	
	HB27 Chrom	1146397	1157468	11071	
T5	HB27 Chrom	129078	129078	0	

	HB27 Chrom	801112	801112	0	
	HB27 Chrom	1285225	1285225	0	
	HB27 Chrom	1315683	1315683	0	
	HB27 Chrom	1525696	1525696	0	
	HB27 pTT27	131276	131276	0	
	HB27 pTT27	150452	150495	43	*hph* down
T6	HB27 Chrom	294855	294855	0	
	HB27 Chrom	296701	299589	2888	*hph* down
	HB27 Chrom	301544	303382	1838	*hph* up
	HB27 Chrom	1281827	1281968	141	
	HB27 Chrom	1302164	1302164	0	
T7	HB27 Chrom	286751	288496	1745	
	HB27 Chrom	290409	290480	71	
	HB27 Chrom	297737	299652	1915	*hph* down
	HB27 Chrom	303310	303958	648	*hph* up
	HB27 Chrom	305805	306069	264	
	HB27 Chrom	307216	307233	17	
	HB27 Chrom	350381	352672	2291	
	HB27 Chrom	354832	356943	2111	
	HB27 Chrom	1161206	1173390	12184	
	HB27 Chrom	1285225	1285225	0	
	HB27 Chrom	1709147	1709147	0	
T8	HB27 Chrom	1835397	1854874	19477	
T9	HB8 Chrom	1499211	1501210	1999	*kat*
	HB8 Chrom	1780464	1780532	68	
T10	HB8 Chrom	637591	637664	73	
	HB8 Chrom	1490912	1493726	2814	
	HB8 Chrom	1499373	1502404	3031	*kat*
	HB8 Chrom	1780464	1780532	68	
T11	HB8 Chrom	1494641	1494641	0	
	HB8 Chrom	1496572	1496581	9	
	HB8 Chrom	1497909	1502722	4813	*kat*
	HB8 Chrom	1780464	1780532	68	
T12	HB8 Chrom	1471034	1501900	30866	*kat*
	HB8 Chrom	1780464	1780532	68	
T13	HB8 Chrom	1499211	1501138	1927	*kat*
	HB8 Chrom	1780464	1780532	68	

1) Single SNPs are reported as 0.

Supplementary Table S2. Strain-specific genes encoded in *T.thermophilus* HB8. Genes present in the *T. thermophilus* HB8 but absent in the related HB27 strain are labelled by their locus name within the four replicons in this strains: the chromosome (TTHAXXX), the megaplasmid pTT27 (TTHBXXX), the plasmid pTT8 (TTHCXXX), and the plasmid pVV8 (TTHVXXX). Putative function of the encoded proteins is provided in brackets.

Chromosome	pTT27 plasmid	pVV8 plasmid	pTT8 plasmid
TTHA0031 (HP)	TTHB003 (HP)	TTHV001 (HP)	TTHC001 (putative RepA protein)
TTHA0032 (HP)	TTHB006 (HP)	TTHV002 (HP)	TTHC002 (HP)
TTHA0033 (HP)	TTHB007 (HP)	TTHV003 (ParA / Cobyrinic acid ac-diamide synthase)	TTHC003 (HP)
TTHA0034 (HP)	TTHB012 (phosphoglycerate mutase family protein)	TTHV005 (helicase domain-containing protein / type III restriction enzyme-2C res subunit)	TTHC004 (HP)
TTHA0035 (HP)	TTHB013 (HP)	TTHV006 (filamentation induced by cAMP protein Fic)	TTHC005 (HP)
TTHA0037 (HP)	TTHB014 (phosphotransferase)	TTHV007 (putative adenine-specific DNA methylase)	TTHC006 (HP)
TTHA0038 (HP)	TTHB015 (putative acyl-CoA dehydrogenase)	TTHV008 (ycfa family protein / HicA)	TTHC007 (HP)
TTHA0150 (HP)	TTHB018 (HP)	TTHV009 (HicB family protein)	TTHC008 (HP)
TTHA0234 (transposase)	TTHB020 (3-oxoacyl-[acyl carrier protein] reductase)	TTHV010 (helix-turn-helix domain protein)	TTHC009 (HP)
TTHA0285 (HP)	TTHB021 (HP)	TTHV011 (HP)	TTHC010 (HP)
TTHA0286 (serine protease)	TTHB026 (HP)	TTHV012 (ATPase (AAA+ superfamily)-like protein)	TTHC011 (HP)
TTHA0478 (trehalose synthase)	TTHB047 (HP)	TTHV013 (HP)	TTHC012 (antitoxin-like protein)
TTHA0479 (trehalose-6-phosphate phosphatase)	TTHB073 (transcriptional regulator)	TTHV015 (LacI-family transcriptional regulator)	TTHC013 (toxin-like protein)
TTHA0480 (trehalose-phosphate synthase)	TTHB084 (transposase)	TTHV016 (phosphonate ABC transporter-2C ATP-binding protein-2C PhnC)	TTHC014 (HP)
TTHA0498 (HP)	TTHB121 (resolvase-related protein)	TTHV017(phosphonate ABC transporter-2C periplasmic phosphonate-binding protein-2C PhnD)	
TTHA0499 (nucleotidyltransferase protein)	TTHB123 (HP)	TTHV018 (phosphonate ABC transporter-2C permease protein-2C PhnE)	
TTHA0640 (HP)	TTHB128 (arsenite oxidase-2C small subunit)	TTHV019 (phosphonate metabolism protein-2C PhnG)	

TTHA0641 (HP)	TTHB129 (transposase)	TTHV020 (phosphonate metabolism protein-2C PhnH)	
TTHA0644 (HP)	TTHB130 (putative transcriptional regulator)	TTHV021 (phosphonate metabolism protein-2C PhnI)	
TTHA0645 (glycosyltransferase)	TTHB133 (HP)	TTHV022 (phosphonate metabolism protein-2C PhnJ)	
TTHA0647 (glycosyltransferase)	TTHB134 (HP)	TTHV023 (phosphonate C-P lyase system protein-2C PhnK)	
TTHA0648 (glycosyltransferase)	TTHB135 (HP)	TTHV024 (phosphonate C-P lyase system protein-2C PhnL)	
TTHA0649 (O-antigen transporter)	TTHB137 (HP)	TTHV025 (phosphonate metabolism protein-2C PhnM)	
TTHA0742 (HP)	TTHB138 (HP)	TTHV026 (probable acetyltransferase-2C phosphonate metabolism protein)	
TTHA0743 (glycosyltransferase)	TTHB139 (major facilitator superfamily permease)	TTHV027 (ABC transporter-like protein)	
TTHA0744 (HP)	TTHB140 (HP)	TTHV028 (extracellular solute-binding protein)	
TTHA0745 (HP)	TTHB141 (putative glycerophosphoryl diester phosphodiesterase)	TTHV029 (metallophosphoesterase)	
TTHA0771 (HP)	TTHB142 (glycerol kinase)	TTHV030 (efflux ABC transporter permease)	
TTHA0772 (HP)	TTHB143 (putative glycerol-3-phohsphate dehydrogenase)	TTHV032 (glycerol-3-phosphate ABC transporter permease)	
TTHA0773 (HP)	TTHB144 (HP)	TTHV033 (sn-glycerol-3-phosphate transport system permease UgpE)	
TTHA0774 (HP)	TTHB168 (HP)	TTHV034 (glycerol-3-phosphate ABC transporter substrate-binding protein)	
TTHA0775 (HP)	TTHB169 (HP)	TTHV036 (glycosyltransferase)	
TTHA0776 (HP)	TTHB170 (HP)	TTHV039 (alkaline phosphatase)	
TTHA0777 (HP)	TTHB173 (response regulator)	TTHV040 (endonuclease/exonuclease/phos phatase)	
TTHA0866 (HP)	TTHB175 (ABC transporter-2C ATP-binding protein)	TTHV041 (HP)	
TTHA0867 (pseudogene)	TTHB176 (putative iron ABC transporter-2C permease protein)	TTHV042 (HP)	
TTHA0932 (HP)	TTHB177 (iron ABC transporter-2C periplasmic iron-binding protein)	TTHV044 (HP)	

TTHA0933 (HP)	TTHB180 (HP)	TTHV045 (HP)	
TTHA1013 (HP)	TTHB185 (HP)	TTHV051 (HP)	
TTHA1014 (HP)	TTHB186 (putative transcriptional regulator)	TTHV052 (HP)	
TTHA1015 (HP)	TTHB187 (HP)	TTHV053 (HP)	
TTHA1016 (HP)	TTHB188 (HP)	TTHV054 (HP)	
TTHA1017 (HP)	TTHB189 (CRISPR-associated Cse2 family protein)	TTHV055 (HP)	
TTHA1018 (transposase)	TTHB190 (HP)	TTHV063 (HP / C-terminus of putative DNA methylase)	
TTHA1020 (HP)	TTHB191 (HP)	TTHV064 (resolvase-2C n terminal domain)	
TTHA1021 (HP)	TTHB192 (HP)	TTHV065 (transposase-2C IS605 OrfB family)	
TTHA1024 (HP)	TTHB193 (HP)	TTHV066 (PilT protein domain protein)	
TTHA1025 (HP)	TTHB194 (HP)	TTHV067 (toxin-antitoxin system-2C antitoxin component-2C PHD family)	
TTHA1051 (HP)	TTHB200 (transposase-like protein)	TTHV069 (HP)	
TTHA1055 (HP)	TTHB201 (transposase-like protein)	TTHV070 (HP)	
TTHA1217 (prepilin-like protein)	TTHB202 (HP)	TTHV073 (HP)	
TTHA1218 (prepilin-like protein)	TTHB232 (transposase)	TTHV074 (HP)	
TTHA1219 (general secretion pathway protein J)	TTHB235 (HP)	TTHV075 (SOS-response transcriptional repressor-2C LexA)	
TTHA1220 (HP)	TTHB237 (2-hydroxymuconic semialdehyde hydrolase)	TTHV076 (protein of unknown function DUF433)	
TTHA1222 (pilin V)	TTHB238 (HP)	TTHV079 (HP)	
TTHA1269 (transposase)	TTHB239 (2-oxopent-4-dienoate hydratase)	TTHV080 (transposase)	
TTHA1325 (sulfite oxidase)	TTHB240 (5-carboxy-2-hydroxymuconate semialdehyde dehydrogenase)	TTHV081 (short-chain dehydrogenase/reductase SDR)	
TTHA1326 (cytochrome C-552 like protein)	TTHB241 (4-oxalocrotonate decarboxylase)	TTHV082 (oxidoreductase domain-containing protein)	
TTHA1336 (peptide ABC transporter substrate-binding protein)	TTHB242 (4-oxalocrotonate tautomerase)	TTHV083 (peptidase M24)	
TTHA1533 (HP)	TTHB243 (HP)	TTHV084 (xylulokinase)	

TTHA1539 (phage integrase/recombina se)	TTHB244 (phenol hydroxylase component B)	TTHV085 (Xylose isomerase)	
TTHA1584 (type II restriction enzyme TthHB8I)	TTHB246 (4-hydroxy-2-oxovalerate aldolase)	TTHV086 (ROK family protein)	
TTHA1646 (HP)	TTHB247 (acetaldehyde dehydrogenase)	TTHV087 (ABC transporter-like protein)	
TTHA1727 (HP)	TTHB248 (IclR family transcriptional regulator)	TTHV088 (permease protein-2C ABC-type xylose transporter)	
TTHA1728 (HP)	TTHB250 (metapyrocatechase (catechol 2-2C3-dioxygenase)	TTHV089 (D-xylose ABC transporter periplasmic substrate-binding protein)	
TTHA1864 (S-layer protein-like protein)	TTHB251 (ABC transporter-2C periplasmic solute-binding protein-related protein)	TTHV090 (transposase)	
TTHA1865 (serine protease)		TTHV091 (transcriptional regulator-2C XRE family)	
TTHA1866 (HP)			
TTHA1867 (transposase)			
TTHA1868 (extracellular serine protease)			
TTHA1871 (HP)			
TTHA1873 (HP)			
TTHA1874 (HP)			
TTHA1905 (HP)			
TTHA1906 (HP)			
TTHA1949 (HP)			

HP refers to hypothetical proteins with yet unknown specific function.

Supplementary Table S3. Strain-specific genes encoded in *T.thermophilus* HB27. Genes present in the *T. thermophilus* HB27 but absent in the related HB8 strain are labelled by their locus name within the four replicons in this strains: the chromosome (TTCXXX), or the pTT27 megaplasmid (TTPXXX). Putative function of the encoded proteins is provided in brackets.

Chromosome (annotation)	Plasmid PTT27 (annotation)
TTC0001 (HP)	TTP0025 (N-acetylglucosamine-6-phosphate deacetylase)
TTC0023 (putative FAD-binding dehydrogenase)	TTP0040 (putative sugar transport protein)
TTC0152 (HP)	TTP0052 (uvsE)
TTC0153 (HP)	TTP0053 (HP)
TTC0154 (nrfC)	TTP0079 (HP)
TTC0155 (thiosulfate reductase precursor)	TTP0080 (HP)
TTC0156 (HP)	TTP0082 (HP)
TTC0157 (two-component response regulator)	TTP0088 (HP)

TTC0158 (two-component sensor)	TTP0089 (HP)
TTC0223 (HP)	TTP0090 (HP)
TTC0273 (transposase)	TTP0091 (cytochrome)
TTC0274 (HP)	TTP0093 (HP)
TTC0277 (HP)	TTP0094 (HP)
TTC0278 (HP)	TTP0098 (HP)
TTC0279 (glycosyltransferase)	TTP0122 (aldehyde ferredoxin oxyrreductase)
TTC0280 (lipopolysaccharide N-acetylglucosaminyltransferase)	TTP0125 (HP)
TTC0281 (HP)	TTP0126 (HP)
TTC0282 (asparagine synthetase)	TTP0127 (HP)
TTC0283 (HP)	TTP0128 (HP)
TTC0284 (pleiotropic regulatory protein)	TTP0132 (HP)
TTC0285 (UDP-N-acetylglucosamine 2-epimerase)	TTP0133 (HP)
TTC0286 (acetyltransferase)	TTP0134 (HP)
TTC0287 (wbpB)	TTP0135 (HP)
TTC0288 (UDP-N-acetyl-D-mannosamine 6-dehydrogenase)	TTP0136 (HP)
TTC0335 (livG)	TTP0139 (HP)
TTC0336 (branched amino acid transport system permease)	TTP0140 (HP)
TTC0337 (livH)	TTP0141 (HP)
TTC0338 (leu, ileu, val, thr and ala binding protein)	TTP0142 (putative plasmid stability protein Y4JK)
TTC0339 (maoC)	TTP0143 (HP)
TTC0340 (putative hydrolase)	TTP0144 (HP)
TTC0341 (putative hydrolase)	TTP0145 (HP)
TTC0342 (acil-CoA ligase)	TTP0149 (HP)
TTC0343 (HP)	TTP0152 (HP)
TTC0372 (serine protease)	TTP0153 (HP)
TTC0397 (L-allo-threonine aldolase)	TTP0158 (HP)
TTC0398 (transcriptional repressor)	TTP0166 (HP)
TTC0402 (HP)	TTP0167 (HP)
TTC0419 (ABC transporter)	TTP0168 (HP)
TTC0420 (ABC transporter)	TTP0170 (dessication protein precursor)
TTC0421 (ABC transporter)	TTP0171 (HP)
TTC0422 (sensory transduction histidine kinase)	TTP0172 (diguanylate cyclase)
TTC0429 (HP)	TTP0174 (HP)
TTC0432 (pmbA)	TTP0175 (ferrichrome binding protein)
TTC0433 (tldD)	TTP0182 (transposase)
TTC0444 (HP)	TTP0185 (HP)
TTC0532 (degV)	TTP0188 (HP)
TTC0563 (cell volume regulation protein CvrA)	TTP0189 (HP)
TTC0575 (HP)	TTP0190 (pilA)
TTC0579 (HP)	TTP0191 (helicase superfamily protein I)
TTC0597 (leu, ileu, val, thr and ala binding protein)	TTP0194 (HP)
TTC0598 (livH)	TTP0210 (transposase)
TTC0599 (livM)	TTP0211 (ATPase)
TTC0600 (livF)	TTP0215 (HP)
TTC0625 (multidrug resistance protein)	TTP0216 (HP)
TTC0626 (marR)	TTP0217 (HP)

TTC0628 (HP)	
TTC0629 (HD hydrolase domain containing protein)	
TTC0635 (HP)	
TTC0636 (exopolyphosphatase)	
TTC0637 (poliphosphate kinase)	
TTC0641 (HP)	
TTC0642 (HP)	
TTC0653 (HP)	
TTC0654 (HP)	
TTC0655 (HP)	
TTC0656 (HP)	
TTC0659 (HP)	
TTC0660 (HP)	
TTC0661 (HP)	
TTC0662 (HP)	
TTC0663 (HP)	
TTC0664 (HP)	
TTC0799 (HP)	
TTC0834 (HP)	
TTC0855 (prepilin-like protein)	
TTC0856 (prepilin-like protein)	
TTC0857 (comZ)	
TTC0858 (pilA)	
TTC0861 (HP)	
TTC0904 (putative esterase)	
TTC0951 (immunogenic protein)	
TTC0952 (transporter)	
TTC0953 (immunogenic protein)	
TTC0961 (sulfite reductase)	
TTC0962 (cytochrome c-522 precursor)	
TTC0971 (dipeptide binding protein)	
TTC0972 (dppB)	
TTC0973 (dppC)	
TTC1088 (protease I)	
TTC1216 (transposase)	
TTC1217 (HP)	
TTC1220 (modification methylase)	
TTC1284 (nucleotidyltransferase)	
TTC1344 (HP)	
TTC1345 (integral membrane protein)	
TTC1364 (HP)	
TTC1365 (HP)	
TTC1370 (lacasse)	
TTC1397 (putative response regulator protein)	
TTC1398 (HP)	
TTC1399 (flavocytochrome C)	
TTC1428 (HP)	
TTC1429 (HP)	
TTC1430 (HP)	
TTC1431 (transposase)	
TTC1432 (transposase)	

TTC1437 (transporter)	
TTC1441 (transporter)	
TTC1472 (HP)	
TTC1497 (HP)	
TTC1499 (HP)	
TTC1513 (sugar-binding protein)	
TTC1532 (S-layer protein)	
TTC1537 (HP)	
TTC1557 (HP)	
TTC1565 (HP)	
TTC1585 (putative amidotransferase)	
TTC1593 (HP)	
TTC1680 (HP)	
TTC1681 (HP)	
TTC1682 (HP)	
TTC1683 (HP)	
TTC1684 (HP)	
TTC1685 (HP)	
TTC1686 (HP)	
TTC1707 (formate tetrahydrofolate ligase)	
TTC1744 (lactate 2-monooxygenase)	
TTC1836 (HP)	
TTC1837 (HP)	
TTC1838 (general secretion pathway protein G)	
TTC1839 (general secretion pathway protein G)	
TTC1840 (HP)	
TTC1841 (HP)	
TTC1842 (HP)	
TTC1843 (HP)	
TTC1844 (putative ATPase)	
TTC1845 (general secretion pathway protein F)	
TTC1846 (HP)	
TTC1877 (restrictase)	
TTC1878 (nurA)	
TTC1879 (tdtA)	
TTC1880 (DNA methylase)	
TTC1881 (DDE transposase)	
TTC1882 (partial transposase)	
TTC1884 (HD hydrolase domain containingcprotein)	
TTC1943 (HP)	
TTC1967 (HP)	
TTC1973 (CBS-domain containing protein)	
TTC1974 (HP)	

HP refers to hypothetical protein with yet unknown specific function.
